# Experimental Study on the Icing Dielectric Constant for the Capacitive Icing Sensor

**DOI:** 10.3390/s18103325

**Published:** 2018-10-04

**Authors:** Yongcan Zhu, Xinbo Huang, Yi Tian, Chao Ji, Wen Cao, Long Zhao

**Affiliations:** School of Electronics Information, Xi’an Polytechnic University, Xi’an 710048, China; zhuyongcan@xpu.edu.cn (Y.Z.); tianyi2017@mail.xjtu.edu.cn (Y.T.); jic2008@nwpu.edu.cn (C.J.); caowen@xpu.edu.cn (W.C.); zhaolong@xpu.edu.cn (L.Z.)

**Keywords:** atmospheric icing, transmission line, icing-detection sensor, dielectric constant, capacitive sensor

## Abstract

The capacitive method is considered to be a suitable icing-detection technology, but the lack of fundamental parameters restricts the development of icing-detection sensors. In this paper, an artificial icing laboratory, a capacitive sensor, and some simulation conductors have been designed for obtaining the artificial icing samples. Subsequently, the same characteristic values of artificial icing have been measured by an LCR device, under a selected frequency. This research found that the value of the icing dielectric constant closely correlated with its density, internal sublayer, and the test temperature. Finally, a fitting formula has been presented for calculating the relative dielectric constant, which may provide some important reference value for the design of icing-detection sensors.

## 1. Introduction

**Figure 1:**
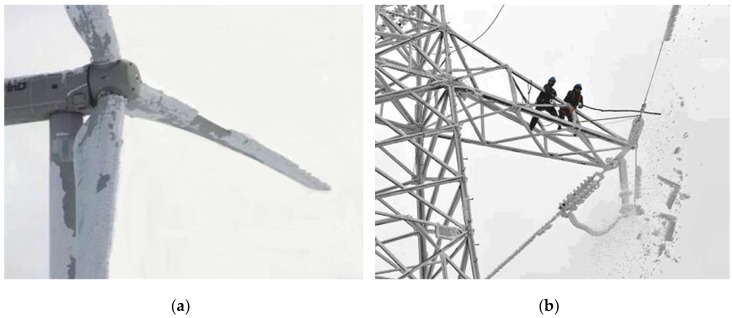
Serious atmospheric icing: (**a**) Icing on wind turbines; (**b**) icing on the transmission line.

Atmospheric icing is a complicated mixture of ice crystal and air, which has affected a wide variety of man-made structures, worldwide, such as overhead transmission lines, wind turbines, and in aircraft [1,2,3,4,5], as shown in Figure 1. In southern Sweden, some turbines were forced to stop working for more than seven weeks, during the best operating period, because a lot of icing had accumulated on turbine blades [1,4]. More seriously, many severe icing disasters on transmission lines have occurred and brought a direct economic loss in Canada, United Kingdom, Iceland, Norway, Russia, China, and some other countries [1,6]. For instance, in the icing disaster that occurred in southern China, in 2008, more than 120,000 transmission towers above 10 kV were damaged, and the direct economic loss was more than twenty billion dollars [6,7].

Atmospheric icing can be distinguished into several forms, by density and appearance, and are named glaze, hard rime, soft rime, wet snow, and hoarfrost [1,2,5]. According to the field observation, the higher the icing density, the stronger the icing adhesion, and the more serious is the resulting security problem [1,5]. In order to mitigate the grave consequences brought by icing disasters, plenty of researchers and engineers have made both theoretical and experimental studies on the icing growth model, de-icing method, anti-icing method, and of course, the icing detection technology [1,2,3,7,8,9,10,11,12,13]. Generally speaking, an effective detection technology is the precondition of the de-icing method [1]. Jiang et al. developed a mechanical calculation model for the equivalent icing load, based on the parameters of tension and the tilt of the insulator strings, at the iced-tangent towers [11]. Huang et al. proposed the image methods, respectively, for automatic icing-detection technology, in which they detected the icing edges and compared the diameter of the transmission conductor before and after icing growth [14,15]. In recent years, optical fiber sensors have been used to monitor temperature, stress, vibration, and other parameters. This special sensor also has a good prospect for applications in transmission line icing-monitoring [16,17,18]. Homola et al. distinguished a total of twenty-nine different icing-detection technology, into direct or indirect methods. They presented the capacitance-type sensor as one of the suitable methods because of its lower power-consumption and weight [4]. Owusu et al. designed a capacitive probe for icing detection and measurement of icing growth-rate in a laboratory, and found that the value of the measurement capacitance correlates with the icing mass, icing thickness, icing density, and the orientation of icing accumulated on probes [12]. Bhattu et al. studied the transient response of the dielectric constant of atmospheric icing, under different operating atmospheric conditions, and found that the icing-detection sensor is feasible, based on the capacitance effect [13,19]. Mughal et al. studied the variations of dielectric property in snow and pure ice, and presented the characteristics of the electrical properties that are affected by density, ambient temperature, conductivity, test frequency, and relaxation time [20,21,22]. These research studies have provided some important theoretical reference for the design of capacitive icing sensors. On one hand, almost all of the test data and numerical results in the literature are based on the conditions of dry snow and pure ice layer, and not natural icing crystals that grow on man-made structures. On the other hand, the empirical equations related to dry snow are not appropriate for high-density icing, such as hard rime and glaze.

The objectives of this study were, (1) to design an experimental method and devices for icing accumulation experiments and icing dielectric constant experiments, and (2) to investigate the relational expression between the icing dielectric constant and its density, which might provide some useful reference for the design of icing-detection sensors.

## 2. The Background of the Capacitive Sensing Technique

### 2.1. The Complex Dielectric Constant

When a time-varying electric field is applied across a parallel plate capacitor, the complex dielectric constant of a given sample can be written as below [19].

(1) $$\varepsilon^{*}=\varepsilon^{\prime }-j\varepsilon^{\prime \prime }=\varepsilon_{\infty }+\frac{\Delta k_{1}}{{\left(1+{\left(j\omega \tau_{1}\right)}^{a_{1}}\right)}^{\beta_{1}}}+\frac{\Delta k_{2}}{{\left(1+{\left(j\omega \tau_{2}\right)}^{a_{2}}\right)}^{\beta_{2}}}-j\frac{\sigma_{0}}{\omega \varepsilon_{0}}$$

where j is the imaginary unit. $\varepsilon^{\prime }$ and $\varepsilon^{\prime \prime }$ denote the real and imaginary part of the complex dielectric constant (F/m). $\varepsilon^{\prime }$ represents the volumetric energy density caused by the external electric field, and $\varepsilon^{\prime \prime }$ represents the dielectric loss factor. $\varepsilon_{\infty }$ denotes the high-frequency dielectric constant (F/m); $\Delta k_{i}$ denote the dispersion strength, and $\tau_{i}$ is relaxation time (s), and i=1,2. $\alpha_{i}$ and $\beta_{i}$ are the factors of dispersion. With different values of $\alpha_{i}$ and $\beta_{i}$, the dielectric dispersion can be defined as Debye-type, Cole-Cole type, and Davidson-Cole type. $\sigma_{0}$ denotes the conductivity of icing samples (S/m). $\varepsilon_{0}$ is the dielectric constant of vacuum (F/m), and ω denotes the angular frequency (rad/s). Takei presented the relaxation time and the Cole-Cole plots of complex dielectric constants for different icing samples, and listed the dielectric properties of the icing samples, along with their different growth processes [19]. These studies have provided some meaningful reference for the icing-detection technology, but the complex dielectric properties are too complex and unavailable are to the detection sensors which are installed in harsh environments. Fortunately, the real part of the complex dielectric constant is enough for icing-detection and icing-type classifications. Therefore, only the real part of the complex dielectric constant of the atmospheric icing samples have been studied here [13].

### 2.2. Mathematical Background for the Experiments on Icing Dielectric Constant

In order to measure the dielectric constant of icing samples, a simulation conductor and two parallel electrodes were designed to obtain icing samples with different growth processes. The capacitance value between the two parallel electrodes can be given by the equation below [13].

(2) $$C_{x}=\frac{\varepsilon_{x}\varepsilon_{0}A}{L}$$

where A and L denote the effective surface area and the distance of the two parallel electrodes ($m^{2}$ and m). $\varepsilon_{0}$ is the dielectric constant of vacuum, $\varepsilon_{0}=8.854\times 10^{-12}\;F/m$. $\varepsilon_{x}$ denotes the relative dielectric constant of the filling material between the two parallel electrodes, and the value of 3.2 and 1.0 are appropriate estimations for pure ice and air [12,21]. When the filling material is an icing sample or no material (air), the capacitance value between the two parallel electrodes can be calculated separately as follows:

(3) $$\left\{ \begin{array}{l} C_{i}=\frac{\varepsilon_{m}\varepsilon_{0}A}{L}\\ C_{f}=\frac{\varepsilon_{f}\varepsilon_{0}A}{L} \end{array}\right.$$

where $\varepsilon_{m}$ denotes the relative dielectric constant of the icing sample. $\varepsilon_{f}$ denotes the relative dielectric constant of air; $\varepsilon_{f}=1$.

### 2.3. The Relative Dielectric Constant for the Two Mixture Materials

Atmospheric icing is a complex mixture of ice crystal and air, and the material proportion can be reflected in its relative dielectric constant [4,12,13]. According to the Wiener relation [20], the relative dielectric constant of a mixed material can be calculated as follows:

(4) $$\frac{\varepsilon_{m}-1}{\varepsilon_{m}+u}=\lambda_{ice}\frac{\varepsilon_{ice}-1}{\varepsilon_{ice}+u}+\lambda_{f}\frac{\varepsilon_{f}-1}{\varepsilon_{f}+u}$$

where $\varepsilon_{ice}$ denotes the relative dielectric constant of pure ice (F/m). $\lambda_{ice}$ and $\lambda_{f}$ denote the volume proportion of pure ice and air, which can be reflected in the parameters of icing density, $\lambda_{ice}=\rho_{m}/\rho_{ice}$, $\lambda_{f}=1-\rho_{m}/\rho_{ice}$. $\rho_{m}$, $\rho_{ice}$ denote the density of the icing sample and pure ice (${\mathrm{kg}/m}^{3}$). Hence, if the dielectric constant of icing sample is known, the icing density and icing type can be accurately estimated, which is extremely meaningful in icing-detection technology. Moreover, u denotes the structural parameter of the icing’s internal sublayer, and it can be valued between 0 and infinity. In the parallel condition of the icing structure model, the ice crystals are assumed to be thin columns, all of which are parallel to the direction of the electric field, written as u→+∞. On the contrary, all of the ice crystals are perpendicular to the direction of the electric field, and this typical icing structure is called a serial icing structure, written as u→0. The parallel and serial structure model represent the maximum and minimum value of u, respectively.

## 3. Experiments

### 3.1. Artificial Icing Laboratory

The natural atmospheric icing growth on transmission lines and wind turbines is highly random and unstable, so it is very difficult to perform an experiment on dielectric constants, with natural icing. In this study, a set of self-design experimental equipment and an artificial icing laboratory have been employed to obtain icing samples with different density and appearance.

**Figure 2:**
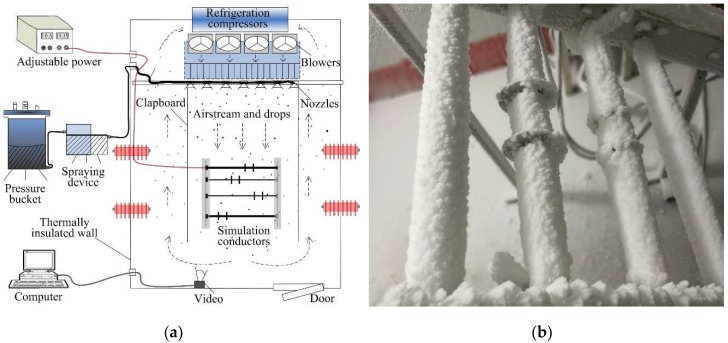
The artificial icing laboratory: (**a**) Schematic diagram of artificial icing laboratory; (**b**) photo of icing samples growth on the simulation conductor.

As shown in Figure 2, the adiabatic laboratory was designed as a 2.2 m × 3.2 m × 4.2 m spatial room with a specific thermally-insulated wall of 15 cm. With two efficient refrigeration compressors, the ambient temperature of the artificial icing laboratory could be accurately controlled within −25~0 °C. Moreover, a pressurized water spray system was employed to adjust the droplets parameters, such as the liquid water mass per unit volume (LWC) and the median volume diameter (MVD), which was composed of an air compressor, pressure bucket, a spraying device, water pipe, and sever nozzles. In addition, several electric fans were positioned in the rear part of the experimental area, which were used to drive the horizontal movement of the water droplets. When the droplets flowed through the simple wind tunnel, the temperature of the microdroplets would reduce rapidly. Thus, the water droplets would move forward, collide with the homemade simulation conductor in a non-uniform manner, and form a significant icing load. Furthermore, the simulation conductors were connected to several controllable low-speed electromotors to ensure that the artificial icing was a standard cylinder. On this basis, a large number of icing samples would be obtained with a different density and internal structure, by changing the experimental conditions, in terms of ambient temperature, wind speed, and droplet parameters.

### 3.2. Experimental Apparatus

**Figure 3:**
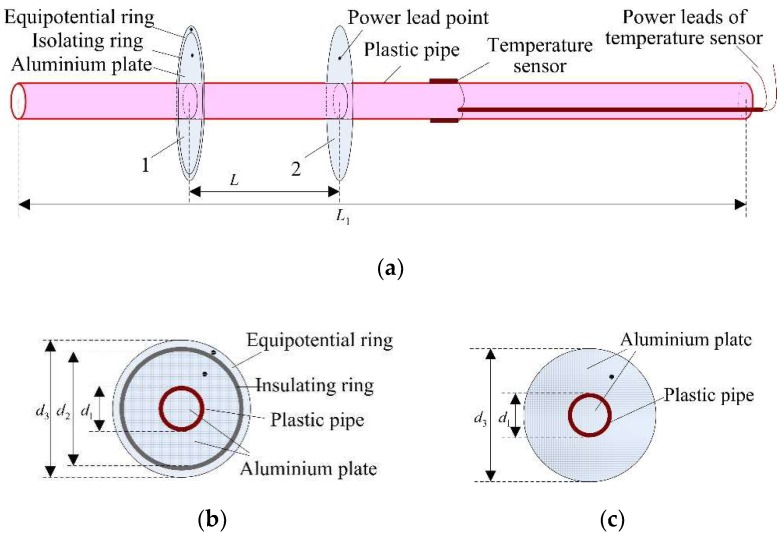
The schematic of the simulation conductor: (**a**) Simulation conductor with two capacitance electrodes; (**b**) no. 1 capacitance electrode; and (**c**) no. 2 capacitance electrode.

As shown in Figure 3a, the homemade simulation conductor was a plastic pipe with a thickness and overall length ($L_{1}$) of 1.5 mm and 0.8 m. Four precision temperature sensors were placed on the outer surface of the plastic pipe. The average reading of the four temperature sensors was assumed to be the real temperature of the inner surface of the icing samples. Two round aluminum plates (capacitive sensors) with the same dimension $d_{3}$ were installed onto the simulation conductor, with a spacing of L. In order to reduce the edge-effect of the capacitive sensor, an isolating ring and an equipotential ring were added outside the No. 1 aluminum plate, which is shown in Figure 3b. Therefore, the cross-section of the capacitance electrode was divided into five parts, called internal aluminum plate, plastic pipe, external aluminum plate, isolating ring, and the equipotential ring. The internal and external aluminum plates were unified, and the plastic pipe was only affixed to the aluminum plate. On the contrary, the equipotential ring and the aluminum plate were completely separated by an insulating plate of 1.5 mm to ensure electrical isolation of the two parts. The diameters of plastic pipe, aluminum electrode, and equipotential ring were $d_{1}$, $d_{2}$, $d_{3}$, respectively. Moreover, as shown in Figure 3c, the No. 2 capacitance electrode was an integral structure with the same diameter of $d_{3}$, and the plastic pipe was pasted onto the aluminum plate, as well.

**Figure 4:**
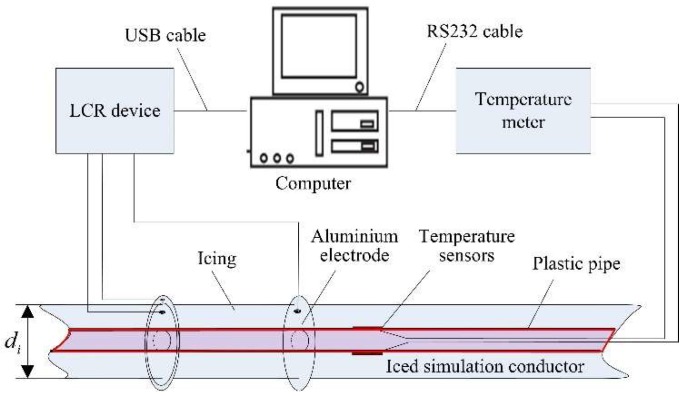
The measurement of the dielectric constant of an icing sample.

When the icing sample diameter $d_{i}$ was close to, or larger than, the diameter $d_{2}$, the icing growth experiment was stopped, and the icing sample was ready. After a quiet period of time, the icing sample was measured for the actual mass and volume. In the measurement process of the dielectric constant, the icing sample was horizontally placed on a wooden bracket. As shown in Figure 4, the capacitance value of the icing sample was measured by an LCR device, which had a variety of selectable test-frequencies between 20 HZ and 2 MHz. Under the selected frequency of 1 MHz, the capacitance values were measured and transferred directly to a computer using a USB cable. It should be noted that, during the capacitance test, the temperature of the iced simulation conductor and the environment was kept below the freezing point. Moreover, the lead wires of the LCR device and the temperature meter was pre-cooled before connecting it to the aluminum electrodes and the temperature sensors.

**Figure 5:**
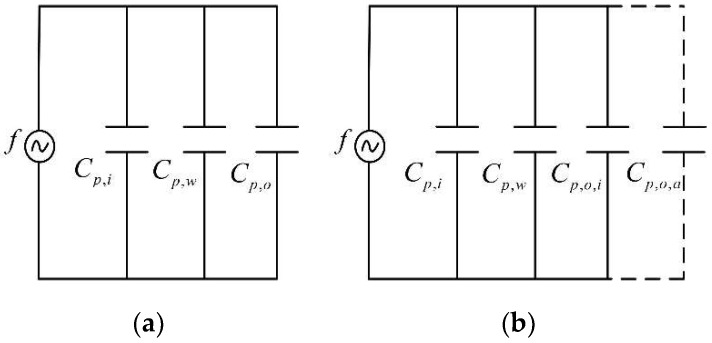
The equivalent circuit of the capacitive sensor, before and after icing accumulation: (**a**) The equivalent circuit before icing accumulation; (**b**) the equivalent circuit after icing accumulation.

As shown in Figure 3, the no-iced parallel plate capacitor could be divided into three parts, the inner hole of the plastic pipe, the plastic pipe wall, and the outside of the plastic pipe. Correspondingly, the capacitance values of the three parts could be expressed, respectively, as $C_{p,i}$, $C_{p,w}$, and $C_{p,o}$. The equivalent circuit of the no-iced parallel plate capacitor was simplified, as shown in Figure 5a. Moreover, after the icing accumulation on the simulation conductor, there was no change in the capacitance values of the pipe’s inner hole and the pipe wall. But the value of $C_{p,o}$ changed to $C_{p,o,i}$, as the medium outside the pipe had changed from air to icing crystal. Additionally, if the diameter of the icing sample was smaller than $d_{2}$, the capacitor could be divided into $C_{p,o,i}$ and $C_{p,o,a}$. These two capacitance values correspond to the iced part and the no-iced part, respectively, as shown in Figure 5b.

In summary, the equivalent capacitance values could be, respectively, written as follows:

(5) $$\left\{ \begin{array}{l} C_{f}=C_{p,i}+C_{p,w}+C_{p,o}\\ C_{i}=C_{p,i}+C_{p,w}+C_{p,o,i}+C_{p,o,a} \end{array}\right.$$

$C_{f}$ and $C_{i}$ denote the measured values of the capacitive sensor, before and after the icing accumulation. Subtracting the two expressions from Equation (5), the differential measured capacitance value could be written as follows:

(6) $$\Delta C_{i}=C_{i}-C_{f}=C_{p,o,i}+C_{p,o,a}-C_{p,o}=\varepsilon_{m}\frac{\varepsilon_{0}A}{L}-\varepsilon_{f}\frac{\varepsilon_{0}A}{L}=\frac{\varepsilon_{0}}{L}(\frac{\pi d_{i}^{2}-\pi d_{1}^{2}}{4})(\varepsilon_{m}-1)$$

Then, the relative dielectric constant of the icing sample could be calculated as below.

(7) $$\varepsilon_{m}=\frac{4L\Delta C_{i}}{\pi \varepsilon_{0}(d_{i}^{2}-d_{1}^{2})}+1$$

### 3.3. Experimental Procedures

The experimental procedures involved the following steps. (1) Laboratory equipment, such as the simulation conductor, the LCR device, and the hotwire anemometer were designed. (2) Weather conditions of the icing growth, such as ambient temperature, airflow velocity, and the droplet parameters were set. (3) The water droplets were allowed to move forward and freeze on the homemade simulation conductor, until the diameter of the icing sample was close to, or larger than, the diameter $d_{2}$. (4) The ambient temperature, was adjusted and maintained, until the reading of the temperature sensor was consistent with the environment. (5) The LCR device lead was connected and the test frequency was adjusted. (6) The measurements of the icing capacitance and the environment temperature were read and recorded. (7) Steps 4–6, were repeated until the number of test data points, of the icing sample, reached *M*. (8) Steps 2–7, were repeated until the amount of icing sample was more than *N*.

## 4. Experimental Results

### 4.1. Icing Crystal Accretion on the Simulation Conductors

**Figure 6:**
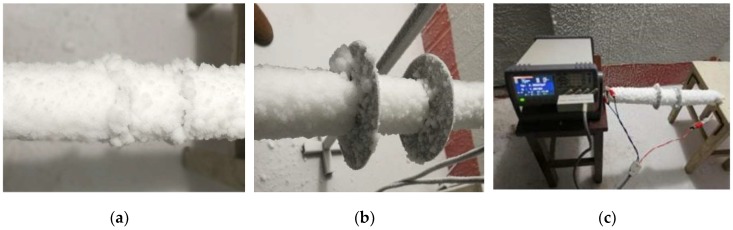
The photos of the different icing sample: (**a**) Soft rime of 24 mm thickness; (**b**) hard rime of 8 mm thickness; and (**c**) measurement of the relative dielectric constant.

The growth type and the growth rate of the atmospheric icing were related closely to the meteorological parameters and the conductor parameters. In the artificial icing laboratory, icing samples with different densities and dielectric constants, could be obtained, these are shown in Figure 6. When the LWC parameter was much smaller and the ambient temperature was lower than −10 °C, there was no extra liquid-drip on the icing surface, since the freezing fraction equaled to 1. In this condition, the icing sample grew into the soft rime. It had a loose structure and fluffy ice trees, as shown in Figure 6a. In contrast to the above-mentioned meteorological parameters, the liquid drop could not be frozen in the collision position, therefore, the icing sample grew into the hard rime or the glaze icing, with a much higher density, as shown in Figure 6b. The surface structures of the hard rime and the glaze were teardrop-shaped, and were much smoother, with a typical roughness-element-height of 1–2 mm. Furthermore, as shown in Figure 6c, after the icing growth experiment, the relative dielectric constant of the icing sample could be measured.

### 4.2. Effects of Exposure Time on the Relative Dielectric Constant of the Icing Sample

**Figure 7:**
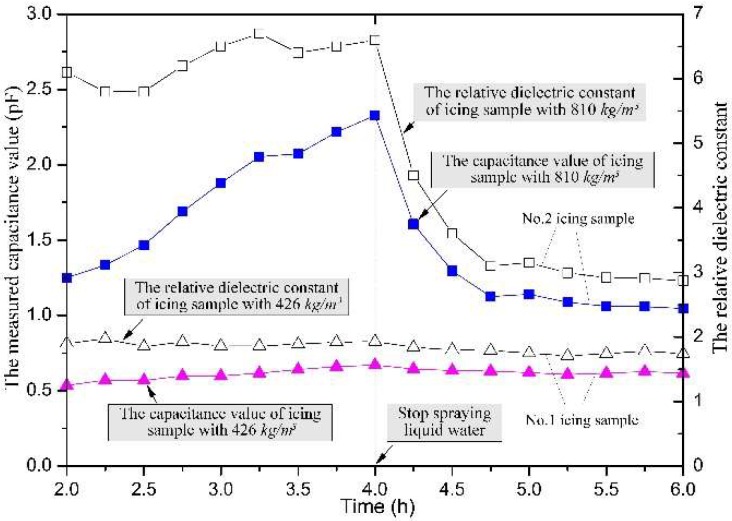
The relative dielectric constant curve of the two icing samples.

The measured capacitance and the relative dielectric constant of the two typical icing samples are shown in Figure 7. The No. 1 icing sample was the result of dry growth, and its final density was only 426 kg/m^3^. The supercooled droplet was frozen locally, at the collision site, and there was no excess droplet flow, so it was assumed that the liquid water content in the icing layer was very low. In the growth process of the soft rime sample, the measured capacitance value increased slowly, with the icing diameter. For comparative analysis, the spray system in the artificial icing laboratory was stopped at 4 h, while the icing accumulation also stopped simultaneously. There was a slight decrease in the measured capacitance value after the time excess of 4 h. Related to this, the relative dielectric constant of the rime icing sample was about 1.95, before 4 h. But after the spray system stopped, the small amount of water in the icing layer was frozen, gradually, and the average temperature of the icing layer gradually decreased to the ambient value. Therefore, the relative dielectric constant of the icing sample also slowly decreased with time, and the value was equal to 1.78, after 1 h.

The second icing sample was the result of wet growth, and its final density was about 810 kg/m^3^. In the process of the icing growth, the supercooled droplet could not be frozen locally, at the collision site, consequently, a thin water film adhered to the icing surface. Therefore, it was assumed that there was a considerable amount of liquid water content in the icing shell. Moreover, because the relative dielectric constant of water is as high as 82, the measured dielectric constant and the capacitance value of the glaze icing sample was much higher than that of pure ice. Accompanied by the icing growth, the measured capacitance value increased quickly with a change in the icing diameter, and the measured capacitance reached a maximum value of 2.23 pF, at the time point when the spraying stopped. After the liquid water-spray stopped, the measured capacitance value decreased rapidly, and gradually stabilized to 1.05 pF, after 5 h. Correspondingly, the relative dielectric constant of the glaze icing sample fluctuated around a value of 6.0, during the icing growth, with a maximal difference value of about 1.0. This significant fluctuation of the relative dielectric constant was mainly caused by the difference in the water content. After the spray system stopped, the water in the glaze sample gradually froze. Thus, the relative dielectric constant of icing sample decreased quickly, and the value stabilized to 2.9, 1 h after the spraying stopped.

This exorbitant measured dielectric constant, in the icing growth process, might create some confusion to the icing-detection sensor. However, the measured capacitance value stabilized to a constant, after 1 h of the icing growth, and this characteristic could also be used to distinguish the icing-growth type.

### 4.3. Effects of Icing Density on Its Relative Dielectric Constant

**Figure 8:**
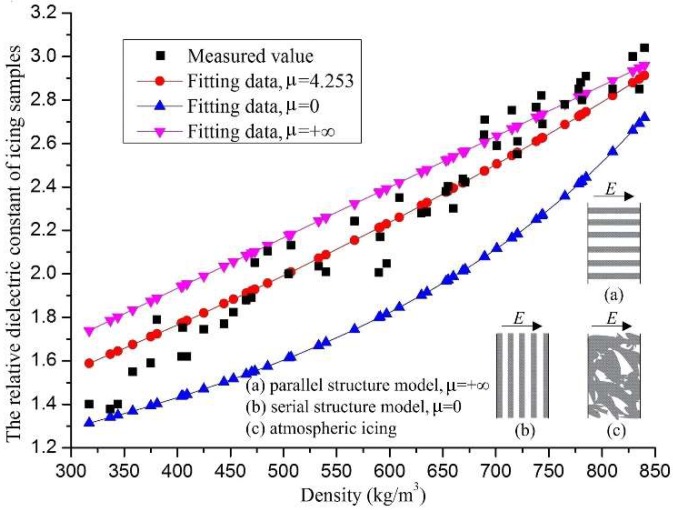
The relative dielectric constant of the icing samples with *μ* = 4.253.

According to Equation (4), the relative dielectric constant of the icing sample is related to its density and internal structure. At the experimental temperature of −10 °C, the relative dielectric constant of the icing samples, with their different densities, are shown in Figure 8, depicted as discrete points. The relative dielectric constant data of the icing samples were dispersive, and there could be a maximum difference of up to 0.25, for the icing samples with the same density. There are three possible reasons for this phenomenon. First, ice crystals grow on capacitor plates, but the degree of tightness between the ice crystals and the capacitor plates could not be controlled. Second, the ice crystal structures inside the icing samples were different. Third, the objects around the capacitor plates had a significant impact on the little-measured capacitance value, so the random error in the measurement process was significant. Furthermore, the general trend was that the relative dielectric constant of the icing sample increased with its density, and the maximum value could reach 3.03, when the density was about 850 kg/m^3^. From Equation (4), the relationship between the relative dielectric constant and the density for the icing samples could be written as below.

(8) $$\frac{\varepsilon_{m}-1}{\varepsilon_{m}+4.253}=\lambda_{ice}\frac{\varepsilon_{ice}-1}{\varepsilon_{ice}+4.253}+\lambda_{f}\frac{\varepsilon_{f}-1}{\varepsilon_{f}+4.253}$$

As shown in Equation (8) and Figure 8, the structural parameter *μ*, of the icing dielectric constant, was fitted as 4.253, and its curve was approximately a straight line with a positive slope. When the density of the icing sample was between 300 and 400 kg/m^3^, the fitting curve was generally larger than that of the experimental data. However, the fitting data was generally smaller than the experimental data when the icing sample density was greater than 700 kg/m^3^. Therefore, it could be considered that there was a significant error of the fitting curve, and Equation (8) was not effective for calculating the relative dielectric constant of the icing samples. In this paper, the icing density was a critical parameter for calculating the icing structural parameter, and the fitting formula of the relative dielectric constant of the icing samples was written as follows:

(9) $$\frac{\varepsilon_{m}-1}{\varepsilon_{m}+(0.0214\rho_{m}-6.504)}=\lambda_{ice}\frac{\varepsilon_{ice}-1}{\varepsilon_{ice}+(0.0214\rho_{m}-6.504)}+\lambda_{f}\frac{\varepsilon_{f}-1}{\varepsilon_{f}+(0.0214\rho_{m}-6.504)}$$

where the icing structural parameter was expressed as *μ* = 0.0214*ρ_m_* − 6.504.

**Figure 9:**
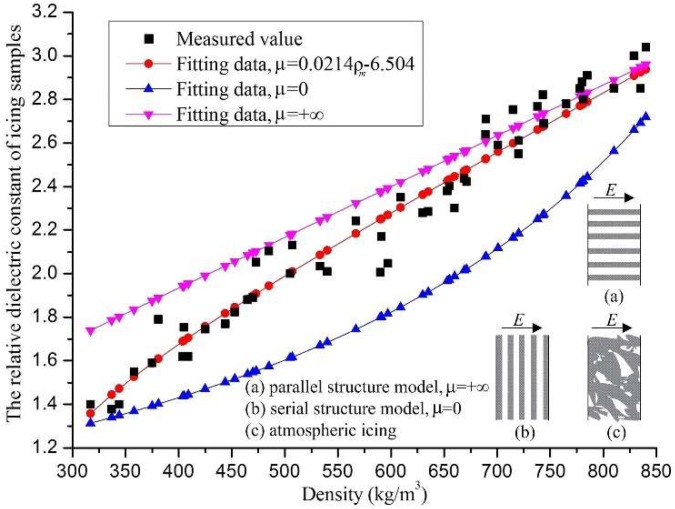
The relative dielectric constant of the icing samples with *μ* = 0.0214*ρ_m_* − 6.504.

As shown in Figure 9, the fitting curve of Equation (9) was, approximately, at the center of the experimental data, and the maximum difference was about 0.21. Under the temperature condition of −10 °C, Equation (9) was appropriate to reflect the relationship between the relative dielectric constant and the density of the icing samples.

### 4.4. Effects of the Icing Temperature on the Relative Dielectric Constant

**Figure 10:**
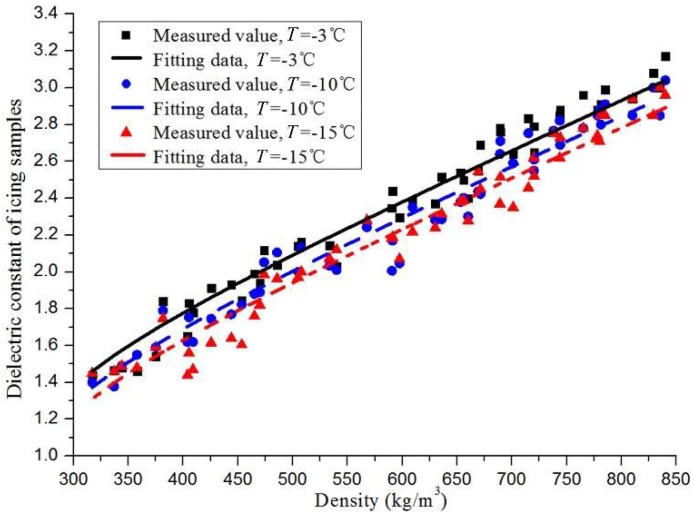
The relative dielectric constant of the icing samples under different temperatures.

Besides the icing density, the temperature also had a direct effect on the relative dielectric constant. At the test temperature of −3, −10, and −15 °C, the experimental relative dielectric constant of the icing samples are depicted as discrete points in Figure 10. The relative dielectric constant of the icing sample increased with the test temperature, and the average difference value was only 0.14, under the test conditions of −3 °C and −15 °C. In the form of Equation (4), the relationship between the relative dielectric constant, the test temperature (*T*), and the density of the icing samples could be fitted as below.

(10) $$\varepsilon_{m}=\frac{\varepsilon_{ice}+(0.0214\rho_{m}-6.504)\left(1+\lambda_{ice}(\varepsilon_{ice}-1)\right)}{\varepsilon_{ice}+(0.0214\rho_{m}-6.504)-\lambda_{ice}(\varepsilon_{ice}-1)}+0.0214T+0.139$$

As shown in Figure 10, the fitting curve of Equation (10) was approximately at the center of the experimental data, in the different temperature conditions. Therefore, this equation reflected the changing trend of the experimental data. Moreover, the experimental values of the relative dielectric constant of the icing samples had an approximate zonal distribution characteristic, at the three test temperatures, although this characteristic was weaker than the data dispersion. The temperature coefficient had a weak effect on the icing dielectric constant, but this equation still had some reference value and guidance significance for research on icing-detection sensors, on wind turbines and overhead transmission lines.

## 5. Conclusions

In this paper, some effective experimental devices were designed and used for icing-parameters measurement. A fitting-formula has been proposed for calculating the relative dielectric constant using a few parameters, such as the icing density, structural parameter, and test temperature.

Although the experimental data were dispersive, the icing density could be regarded to be a major influential parameter of the relative dielectric constant, and the value of the relative dielectric constant increased with an increase in the icing density.

The measured relative dielectric constant of the icing samples had an approximate zonal distribution characteristic, at specific test temperatures. The relative dielectric constant of the icing sample increased with the test temperature, and the average difference value was about 0.14, under the test condition of −3 and −15 °C.

The measured relative dielectric constant of the growing icing sample was significantly higher than the stabilized ones, especially for the glaze-icing sample. This phenomenon was mainly caused by the liquid water content, which might be used to distinguish the icing-types, between glaze and rime.
